# Distribution of the *FYB*^*ES *^and RHCE*ce(733C>G) alleles in an Argentinean population: Implications for transfusion medicine

**DOI:** 10.1186/1471-2350-9-40

**Published:** 2008-05-06

**Authors:** Carlos M Cotorruelo, Silvana V Fiori, Silvia E García Borrás, Liliana L Racca, Claudia S Biondi, Amelia L Racca

**Affiliations:** 1 Laboratorio de Inmunohematología e Inmunogenética, Facultad de Ciencias Bioquímicas y Farmacéuticas, Universidad Nacional de Rosario, Suipacha 531, 2000 Rosario, Argentina; 2 CONICET, Argentina; 3 Área Estadística y Procesamiento de Datos, Facultad de Ciencias Bioquímicas y Farmacéuticas, Universidad Nacional de Rosario, Suipacha 531, 2000 Rosario, Argentina

## Abstract

**Background:**

The understanding of the molecular bases of blood groups makes possible the identification of red cell antigens and antibodies using molecular approaches, especially when haemagglutination is of limited value. The practical application of DNA typing requires the analysis of the polymorphism and allele distribution of the blood group genes under study since genetic variability was observed among different ethnic groups. Urban populations of Argentina are assumed to have a white Caucasian European genetic component. However, historical and biological data account for the influence of other ethnic groups. In this work we analyse *FY *and *RH *blood group alleles attributed to Africans and that could have clinical implications in the immune destruction of erythrocytes.

**Methods:**

We studied 103 white trios (father, mother and child, 309 samples) from the city of Rosario by allele specific PCRs and serological methods. The data obtained were analysed with the appropriate statistical test considering only fathers and mothers (n = 206).

**Results:**

We found the presence of the FY*B^*ES *^and RHCE*ce(733C>G) alleles and an elevated frequency (0.0583) for the Dce haplotype. The number of individuals with a concomitant occurrence of both alleles was significantly higher than that expected by chance. We found that 4.68% of the present gene pool is composed by alleles primarily associated with African ancestry and about 10% of the individuals carried at least one *RH *or *FY *allele that is predominantly observed among African populations. Thirteen percent of Fy(b-) subjects were FY*A/FY*B^*ES*^.

**Conclusion:**

Taken together, the results suggest that admixture events between African slaves and European immigrants at the beginning of the 20^th ^century made the physical characteristics of black Africans to be invisible nowadays. Considering that it was a recent historical event, the FY*B^*ES *^and RHCE*ce(733C>G) alleles did not have time to become widespread but remain concentrated within families. These findings have considerable impact for typing and transfusion strategy in our population, increasing the pool of compatible units for Fy(b-) individuals requiring chronic transfusion. Possible difficulties in transfusion therapy and in genotyping could be anticipated and appropriately improved strategies devised, allowing a better management of the alloimmunization in the blood bank.

## Background

Classical haemagglutination is a powerful technique for testing blood group antigens but has certain limitations. The understanding of the molecular bases associated with blood group antigens makes possible the prediction of phenotypes using molecular approaches [1-3]. However, the practical application of DNA typing requires an exhaustive analysis of the polymorphism and allele distribution of the blood group genes under study since a high level of genetic variability was observed among different ethnic groups [4-13]. DNA typing in random samples without reference to the allele pool involved may lead to erroneous results.

Many blood group antigens are the result of single nucleotide polymorphisms (SNPs) inherited in a straightforward Mendelian manner. The Duffy (*FY*) blood group locus, localized on chromosome 1q22-q23, is characterized by two major codominant alleles designed FY*A and FY*B. Both alleles are distinguished by a missense mutation (125G>A), which results in a single amino acid difference (Gly42Asp) and gives the common Fy(a+b-), Fy(a-b+) and Fy(a+b+) phenotypes in European and Asian populations [14-18]. The Fy(a-b-) phenotype is commonly found in Blacks homozygous for a silent FY*B allele which is caused by a substitution from T to C at the GATA box motif of the FY*B promoter (-33 t>c). This mutation disrupts the binding site for the GATA-1 erythroid transcription factor resulting in the lack of *FY *gene expression only in the erythroid linage [19,20]. We refer to this allele as FY*B^*ES *^(ES stands for erythroid silent) [21].

The *RH *blood group locus is localized on the short arm of chromosome 1p34-p36 and its alleles and haplotypes show substantial ethnic variability. This is particularly demonstrated by the RHCE*ce(733C>G) allele that generates the VS antigen which is extremely rare in people of European and Asian origin but has a frequency up to 50% in African descents [10,12,22]. RHCE*ce(733C>G) results from a single point mutation in *RHCE *exon 5 (733C>G), leading to a Leu245Val substitution and has been primarily found in the Dce haplotype [23-25] which is also more common in Blacks with a frequency of 40–60% [9].

Urban populations of Argentina are assumed to have a predominantly white Caucasian European genetic component as a consequence of the massive immigration from Spain and Italy at the beginning of the 20^th ^century [26]. However, diverse ethnic-historic sources consider the Argentinean population to be a hybrid of Europeans, Amerindians and Africans [27]. Recent biological information revealed the presence of approximately 20% of an Amerindian genetic component among Argentineans from different cities of the country [28-30]. As regards the African influence, historical data establish up to 30% of individuals of African origin living in Buenos Aires during most of the 19^th ^century but it still remains controversial among historians whether they disappeared from the population without living descents or was the admixture with the European immigrants that made the physical characteristics of black African people imperceptible nowadays [31]. Few genetic studies have examined the African contribution to the genetic pool of the Argentinean population [29,30,32], hence further analysis are needed. In this work we performed a random survey among white individuals from the city of Rosario to describe the presence of blood group alleles attributed to sub-Saharan Africans. The results contribute to understand the genetic background of our population, thus helping develop reliable high-throughput platforms for molecular blood group typing. Thereby, our findings have important implications in transfusion medicine allowing a better management of the alloimmunization in the blood bank and improving transfusion outcomes.

## Methods

### Blood samples

We studied EDTA blood samples drawn from 103 trios (father, mother and child) from the city of Rosario that concurred to our laboratory for paternity testing. They were chosen in consecutive order considering only those cases in which the paternity was probed by STR analyses. Fathers and mothers filled in a form with personal data. In this form they were also asked to indicate what racial/ethnic group they identified with: 1) White, 2) Black, 3) Amerindian, 4) Asian and 5) other. All parents identified themselves as Whites. The 309 samples were taken with the informed consent of the patients and all procedures were performed according to the ethical standards established by the University of Rosario. Saline erythrocyte suspensions were used for serological studies. Genomic DNA isolated with a commercial kit (QIAamp, Qiagen, Germany) was used for molecular analyses.

### Detection of *FY *alleles

*FY *genotyping was performed by PCR strategies with oligonucleotide primers designed with the allele-differentiating base at the 3' position [33]. Two PCR reactions each containing a forward primer (nucleotide -33T at the 3' end) to target the normal *FY *alleles' promoter region paired with reverse primers specific to anneal FY*A and FY*B (125C and 125T at the 3' end) alleles respectively were set up. The oligonucleotide sequences of each primer used in this study (Operon Biotechnologies, Germany) and the PCR products length are listed in Table 1. The FY*B^*ES *^allele was detected with a forward primer that anneal the mutated promoter region (-33C at the 3' end) paired with the FY*B specific primer.

**Table 1:** Sequences of primers and PCR products length

Analysis of	Primers	5'-3' nucleotide sequence^a^	Product length
*FY *alleles	Fy normal 2	ccctcattagtccttggctcttct	713 bp
	Fy null	ccctcattagtccttggctctttc	
	FyA4	CAGCTGCTTCCAGGTTGGCTC	
	FyB4	CAGCTGCTTCCAGGTTGGCTT	

Hybrid *Rhesus box*	5' IR	tcctgcagcaaacttctga	1980 bp
	3' IR	tctcttttctggccttaacatc	

RHCE*ce(733C>G) allele	RHCEint4F1	agactgctgggagaggctaat	807 bp
	733G rev	CACCACGCTGACTGCTAC	
	733C rev	CACCACACTGACTGCTAG	

HLA Class II *DRB1 *exon 2	2DRBAmpA	ccgaccacgacgtttcttg	274 bp
	2DRBAmpB	ccgctgcactgtgaagctctc	

a. Capital letters denote nucleotides in exons while small letters denote nucleotides in non-coding sequences. Intentionally mismatched bases are underlined.

### Detection of Dce haplotypes

Routine erythrocyte Rh antigens' typing was performed by standard agglutination methods using polyclonal as well as monoclonal antibodies against D, C, c, E and e (DiaMed, Switzerland). Haplotypes were inferred in each individual by the most probable genotype method using standard frequency tables for Whites. *RHD *zygosity was also determined by *Rhesus box *analysis. A copy of a hybrid *Rhesus box *was detected by a PCR strategy [25] using a forward primer that anneals at the 5' end of the identity region of the upstream and hybrid *Rhesus boxes *(specific for 5465A) and a reverse primer that anneals at the 3'end of identity region of the downstream and hybrid *Rhesus boxes *(specific for 7403G) (Table 1).

### Detection of RHCE*ce(733C>G) alleles

Two separate PCR were performed using a forward primer specific for intron 4 of the *RHCE *gene and reverse primers complementary to *RH *exon 5 containing at their 3' ends the polymorphic nucleotides 733G (to anneal in *RHCE *exon 5) or 733C (to anneal in *RHD *exon 5) in each reaction [25] (Table 1).

### PCR conditions

All PCR reactions contained a pair of primer to amplify exon 2 of the *DRB1 *gene as internal positive control [34]. Amplifications were performed in a thermal cycler (PTC-200 MJ Research, Waltham, MA) with approximately 0.5 μg of genomic DNA in a final volume of 20 μl containing 0.4 μM of each primer (except for primers used for internal positive controls that were at 0.04 μM), 0.2 mM of each dNTP, 2 mM MgCl_2 _and 1 unit of Taq DNA polymerase (Promega, Madison, WI) in appropriate buffer. PCRs started with one cycle of denaturation at 94°C for 2 minutes and were ended with one cycle of 15 minutes at 72°C to complete extension. Cycling parameters were 30 cycles of 30 seconds at 94°C, 1 minute at 59°C (for hybrid *Rhesus box*) or 60°C (for RHCE*ce(733C>G) and *FY *alleles) for annealing and 3 minutes (for hybrid *Rhesus box*) or 1 minute at 72°C for extension. PCR products were analysed by electrophoresis on 1% (for hybrid *Rhesus box*) and 2% agarose gels stained with ethidium bromide.

### Statistical analyses

Allele and haplotype frequencies were obtained by direct counting analysing only fathers and mothers (n = 206). The haplotype frequencies obtained were corroborated by the maximum likelihood method. The chi-square (goodness of fit) test was applied to compare the frequency distribution of *FY *alleles and *RH *haplotypes with those reported. Each proportion individually against the corresponding published value was compared by means of the z test. The fit of genotype frequencies to Hardy-Weinberg proportions was assessed using the chi-square test. Alleles association was analysed by the Fisher's exact test. African admixture was calculated using the gene identity method [35], implemented in the Admix95 program (see Availability and requirements section for URL) and using allele frequencies reported in specialized immunohaematology journals (Tables 2 and 3).

## Results

### Detection of *FY *alleles

Allele and genotype frequencies are shown in Table 2. The frequencies of the three alleles differed significantly from those reported for Whites by molecular methods [7]. An increased in the frequency of the FY*A and FY*B^*ES *^alleles was found while the FY*B allele frequency was reduced (p < 0.005). Fifteen individuals of the 206 fathers and mothers analysed carried the FY*B^*ES *^allele and all of them were heterozygous.

**Table 2:** *FY *allele and genotype frequencies

Genotypes	N° of individuals (total = 206)	Frequency^a^	Whites^b^	Blacks^b^
FY*A/FY*A	60	0.2913	0.1900	0.0000
FY*A/FY*B	80	0.3883	0.3800	0.0200
FY*B/FY*B	51	0.2476	0.4100	0.0200
FY*A/FY*B^*ES*^	9	0.0437	0.0200	0.2600
FY*B/FY*B^*ES*^	6	0.0291	0.0000	0.0900
FY*B^*ES*^/FY*B^*ES*^	0	0.0000	0.0000	0.6100

Alleles	N° of alleles (total = 412)	Frequency^a^	Whites^b^	Blacks^b^

FY*A	209	0.5073	0.3950	0.1000
FY*B	188	0.4563	0.5950	0.1000
FY*B^*ES*^	15	0.0364	0.0100	0.8000

a. Allele and genotype distributions were consistent with the Hardy-Weinberg expectations.

b. Data reported in reference 7.

### Determination of Dce haplotypes

The *RH *haplotypes harboured by each individual was assigned considering the CcEe phenotype and the *RHD *zygosity determined by PCR. The absence of a hybrid *Rhesus box *was indicative of *RHD *homozygosity. If different combinations of haplotypes could be assigned to a given sample, the combination requiring the least number of infrequent haplotypes was chosen as the most plausible explanation (Table 3). Most haplotype frequencies did not differ significantly from those published in the literature for Caucasians. An increased in the frequency of DcE was found (p < 0.05). Serological and molecular *Rhesus box *analyses allowed the detection of the Dce haplotype not only in Dccee (Dce/dce) but also in DCcee (DCe/Dce), DccEe (DcE/Dce) and Dccee (Dce/Dce) phenotypes (Table 3). These findings resulted in an increase in the frequency of the Dce haplotype (p < 0.0005) when compared with those published in the literature for Whites [36] (Table 4).

**Table 3:** Serologic and molecular study of the Rh system

Phenotype	HRB^a^	C733	G733	Genotype^b^	N° of individuals (total = 206)	Frequency
DCcee	+	+	-	DCe/dce	69	0.3350
	+	+	-	Dce/dCe	1	0.0049
	-	+	-	DCe/Dce	4	0.0194
	-	+	+	DCe/Dce(733C>G)	2	0.0097

DCCee	-	+	-	DCe/DCe	28	0.1359
	+	+	-	DCe/dCe	1	0.0049

DCcEe	-	+	-	DCe/DcE	26	0.1262
	+	+	-	DCE/dce	1	0.0049
	+	+	-	DcE/dCe	1	0.0049

DccEe	+	+	-	DcE/dce	17	0.0825
	+	+	-	Dce/dcE	1	0.0049
	-	+	-	DcE/Dce	3	0.0146
	-	+	+	DcE/Dce(733C>G)	3	0.0146

DccEE	-	+	-	DcE/DcE	10	0.0485

Dccee	+	+	-	Dce/dce	5	0.0243
	+	+	+	Dce(733C>G)/dce	3	0.0146
	-	+	+	Dce/Dce(733C>G)	1	0.0049

DCcEE	-	+	-	DcE/DCE	1	0.0049

DCCEe	-	+	-	DCe/DCE	1	0.0049

dccee	+	+	-	dce/dce	25	0.1214

dCcee	+	+	-	dCe/dce	3	0.0146

a. HRB = Hybrid *Rhesus box*. "+" means "presence" while "-" means "abscence".

b. *RH *haplotypes in the different Rh phenotypes were determined considering the CcEe phenotype, the *RHD *zygosity determined by PCR and the segregation analysis of the Rh antigens in each trio. In those cases in which different combinations of haplotypes could be assigned to a given sample, the combination requiring the least number of infrequent haplotypes in both parents was chosen as the most plausible explanation.

**Table 4:** *RH *haplotype frequencies

Haplotypes	N° of haplotypes (total = 412)	Frequency^a^	Whites^b^	Blacks^b^
DCe	159	0.3859	0.4205	0.0602
DcE	71	0.1723	0.1411	0.1151
Dce	24	0.0583	0.0257	0.5908
DCE	3	0.0073	0.0024	0.0000
dce	148	0.3592	0.3896	0.2028
dCe	6	0.0146	0.0119	0.0311
dcE	1	0.0024	0.0098	0.0000
dCE	0	0.0000	0.0000	0.0000

a. Haplotype distributions were consistent with the Hardy-Weinberg expectations.

b. Data reported in refernce 36.

### Detection of the RHCE*ce(733C>G) allele

PCR studies revealed the presence of RHCE*ce(733C>G) in 9 samples of the 206 fathers and mothers studied (Table 3). Although not typed serologically, it can be inferred that the VS antigen has a frequency of 4.37% in the population analysed. None of the samples were homozygous for RHCE*ce(733C>G). The allele frequency is shown in Table 5.

**Table 5:** RHCE*ce(733C>G) allele frequency

Allele	N° of alleles (total = 412)	Frequency^a^	Whites^b^	Blacks^b^
RHCE*ce(733C>G)	9	0.0218	0.0000	0.2800

a. Allele distribution was consistent with the Hardy-Weinberg expectations.

b. Data reported in reference 10.

### Concomitant occurrence of FY*B^ES ^and RHCE*ce(733C>G) alleles

We further analysed how likely it was to observe in individuals harbouring the *FYB*^*ES *^allele the presence of the RHCE*ce(733C>G) allele. The results are shown in Table 6. We found 3 individuals with both FY*B^*ES *^and RHCE*ce(733C>G) alleles. The Fisher's exact test showed a FY*B^*ES*^/RHCE*ce(733C>G) association (p = 0.0203). Moreover, if the presence of the RHCE*ce(733C>G) allele were totally independent of the presence of the FY*B^*ES *^allele, we might expect that the probability for one individual to possess both alleles by chance is 0.0031 in the population analysed. This value indicates that we would not find any individual with both alleles in 206 samples but in fact we found 3. The number of individuals FY*B^*ES *^and RHCE*ce(733C>G) positive was significantly higher than that expected (p < 0.0001, hypothesis test concerning the rate parameter of a Poissson distribution). Segregation analysis within each family group showed no linkage disequilibrium between *FY *and *RH *loci. Rather, the concomitant occurrence of these two alleles may be due to African ancestry in the population analysed.

**Table 6:** Concomitant occurrence of FY*B^*ES *^and RHCE*ce(733C>G)

FY*B^*ES*^	Negative	Positive	Total
RHCE*ce(733C>G)			
Negative	185	12	197
Positive	6	3	9
Total	191	15	206

### Genetic admixture

The value of the genetic admixture with Africans indicates that 4.68% of the present gene pool is composed by alleles primarily associated with African ancestry. Even though the level of admixture is low, we found that approximately 10% of the sample account for it since 21 of the 206 parents have at least one *RH *or *FY *allele that is predominantly observed among African populations (Table 6).

## Discussion

Transfusion medicine is poised to take a leadership role in the large-scale implementation of human genotyping. It already manages mass-scale programs to detect viral contaminants of blood products via nucleic acid technology regimes. This existing infrastructure within blood centres together with the knowledge of the molecular bases of blood groups is being exploited for the development of automated high-throughput microchip technology to simultaneously determine multiple blood group alleles on one sample. However, studies need to be conducted into the genetic variability and distribution of blood groups among different populations to assure a strict genotype – phenotype correlation and to develop reliable genotyping strategies [1-3]. In this work we performed a random survey among 309 white individuals from Rosario, the third largest city of Argentina, to describe the presence of blood group alleles that are typically found in Africans and could have clinical implications in immune haemolysis. We studied the Duffy and Rh systems by reliable serological and molecular methods. We also determined the *RHD *zygosity by detection of a hybrid *Rhesus box *using a PCR strategy that proved to be accurate for testing our population [25].

The molecular analysis of the Duffy and Rh systems reveal that the genetic pool analysed is composed by Caucasian and non-Caucasian alleles. The elevated frequencies found for the FY*A allele and the DcE haplotype (Tables 2 and 4) accounts for the Native American genetic contribution [28]. These results support the still debated New World migration model through the Bering Strait [37,38] since both South American Amerindians and Asian populations are characterized by a prevalence of this *FY *allele and *RH *haplotype [22].

On the other hand, the detection of FY*B^*ES *^and RHCE*ce(733C>G) and the elevated frequency of Dce (Tables 2 and 4) can be attributed to the African influence. These findings are consistent with historical data that establish that by the beginning of the 19^th ^century a third of the population of Argentina was of black race, most of them slaves brought from West Africa. The number of Afro-Argentine individuals diminished dramatically by the end of that century being the current population characterised by the absence of people with morphologically detectable African ancestry [31]. Although the causes that led to the drastic reduction of Blacks remain controversial, our findings among Whites of alleles that are highly frequent in Africans account for the contribution of admixture events. The low number of individuals with FY*B^*ES*^, RHCE*ce(733C>G) and Dce and the 4.68% of African admixture suggest that a small number of African slaves that were living in the Rosario region mixed with the European immigrants that came at the beginning of the 20^th ^century making, after approximately three generations, the physical characteristics of black Africans invisible. This African contribution is somewhat greater than that found in Buenos Aires (2.2%, n = 90) [32] and also higher than the 1.7% obtained studying 88 individuals [30] from Buenos Aires (n = 15), Córdoba (n = 33), Santa Fé (n = 33), Mar del Plata (n = 11) and La Plata (n = 2) by the analysis of different SNPs but lower to that found in the population of La Plata (6.5%, n = 87) [29] by mithocondrial DNA and Y-chromosome-specific sequences studies. Although these results show that in urban populations of Argentina there is a low African ancestry inclusion, we found that 10% of the individuals from Rosario have at least one of the two alleles that are typically found in Africans. This genetic study accounts for the extent of the African influence suggesting that the African people living in Rosario did not disappear but just faded into the mixed-race populace and became lost to demography as immigration exploded. In addition, we found a higher frequency of concomitant occurrence of the FY*B^*ES *^and RHCE*ce(733C>G) alleles per individual than expected by chance (Table 6). This result clearly shows a non-random association between them suggesting that these alleles did not have time to become widespread in the population, but tend to remain concentrated within families. Further analyses of genetic markers could be performed to determine more accurately the African influence in the city of Rosario. Linkage disequilibrium due to admixture between *FY *and *RH *loci could be discarded not only because both loci are located very far apart in chromosome one but also because of segregation analysis within each family group. The African ancestry in the population analysed could explain the significant association found between FY*B^*ES *^and RHCE*ce(733C>G).

Molecular study of the Duffy system showed that 13% (9/69) of Fy(b-) individuals are FY*A/FY*B^*ES *^(Table 2). This finding has implications for the management of transfusion therapy in this population because such patients express normal levels of the FY*B product in tissues other than red blood cells [19,20]. Thus, they would not mount an immune response if they are exposed to Fy(b+) erythrocytes and therefore may not need Fy(b-) blood for transfusion. *FY *genotyping would allow increasing the pool of compatible units, mainly benefiting those requiring chronic transfusions. Based on our findings, we recommend the use of PCR to further characterize these patients and accurately distinguish FY*A/FY*A from FY*A/FY*B^*ES *^so as to implement a more rationale use of available blood units.

The frequency of the RHCE*ce(733C>G) allele (0.0218) allowed us to deduce that the VS antigen is present in approximately 4% of the population. This antigen is immunogenic and could potentially be involved in the haemolytic disease of the foetus and newborn or transfusion reactions [36]. VS is not on commercially available panels red blood cells, we therefore propose to test sera suspected to have alloantibodies with VS+ erythrocytes so that anti-VS may not go undetected thus ensuring transfusion safety and adequate prenatal care.

## Conclusion

In conclusion, in this work we show the contribution of molecular immunohaematology to detect alleles that may be involved in the immune destruction of erythrocytes. We analysed two blood group alleles that are primarily associated with African ancestry by rapid and accurate allele specific PCRs and standard agarose gel electrophoresis which can easily be implemented in laboratories performing basic molecular biology techniques without the need of sophisticated equipment. Genetic data obtained in this study also provide information that help understanding the history of our population, complementing and expanding the evidence that can be gathered from other sources. These results challenge assumptions on a mainly European origin and reveal a more multi-ethnical identity of the population in the Rosario region of central Argentina. Taken together, the results obtained could have considerable impact for typing and transfusion strategy in our population, possible difficulties in transfusion therapy and in genotyping could be anticipated and appropriately improved strategies devised, allowing a better management of the alloimmunization in the blood bank.

## Availability and requirements

Admix95:  

## Abbreviations

PCR: polymerase chain reaction; EDTA: ethilendiaminetetraacetate; STR: short tandem repeats.

## Competing interests

The authors declare that they have no competing interests.

## Authors' contributions

CMC and ALR conceived and designed the study. CMC carried out the molecular genetic studies, analyzed and interpreted the data and drafted the manuscript. ALR analyzed and interpreted the data and drafted the manuscript. SVF collaborated with DNA isolation and molecular genotyping. SEGB and CSB collected the samples and carried out the immunohaematological assays. LLR performed the statistical analyses. All authors read and approved the final manuscript.

## Pre-publication history

The pre-publication history for this paper can be accessed here:


